# Thyroid-stimulating hormone elevation misdiagnosed as subclinical hypothyroidism following non-convulsive status epilepticus: a case report

**DOI:** 10.1186/1752-1947-5-432

**Published:** 2011-09-05

**Authors:** Akira Wada, Yoshiaki Suzuki, Sanae Midorikawa, Satoshi Takeuchi, Yasuto Kunii, Hirooki Yabe, Shin-Ichi Niwa

**Affiliations:** 1 Department of Neuropsychiatry, Fukushima Medical University School of Medicine, Fukushima, Japan; 2 Shimizu Hospital, Fukushima, Japan; 3 Department of Nephrology, Hypertension, Endocrinology and Metabolism, Fukushima Medical University School of Medicine, Fukushima, Japan

## Abstract

**Introduction:**

Non-convulsive status epilepticus is a form of epileptic seizure that occurs without convulsions. Recent reviews suggest that the diagnosis of non-convulsive status epilepticus remains difficult. Here, we report the case of a patient with thyroid-stimulating hormone elevation misdiagnosed as subclinical hypothyroidism following non-convulsive status epilepticus.

**Case presentation:**

Our patient was a 68-year-old Japanese woman. The results of endocrine testing after her first episode of non-convulsive status epilepticus suggested latent subclinical hypothyroidism: she had elevated thyroid-stimulating hormone with normal levels of free tri-iodothyronine and free thyroxine. On examination, a diagnosis of thyroid disorder was not supported by other test results and our patient remained untreated. A follow-up examination revealed that her thyroid-stimulating hormone levels had spontaneously normalized. When she consulted another doctor for confusion, the transient increase in thyroid-stimulating hormone levels following non-convulsive status epilepticus was mistaken for subclinical hypothyroidism, and unfortunately treated with levothyroxine. Our patient then experienced levothyroxine-induced non-convulsive status epilepticus.

**Conclusions:**

In this report, we suggested possible mechanisms for latent hypothyroid-like hormone abnormality following epileptic seizures and the possibility of provoking epileptic seizures by administering levothyroxine for misdiagnosed subclinical hypothyroidism.

## Introduction

Non-convulsive status epilepticus (NCSE) is a condition primarily diagnosed through electroencephalography (EEG). The differentiation of NCSE from other neurological and psychiatric disorders is necessary because of possible damage to many high-order functions including consciousness, emotion, thinking, and memory. Absence epilepsy was first described by Lennox in 1945 [1], and Gastaut and Roger reported non-convulsive complex partial status epilepticus in 1956 [2]. Since then, these seizures and the associated neurological symptoms have been found to be linked, and it is now possible to determine whether they are paroxysmal or non-paroxysmal. A recent review suggested that the diagnosis of NCSE remains difficult, and a high index of suspicion is required to recognize and correctly diagnose this illusive condition [3]. NCSE is a rare condition, reportedly affecting only 38 people per 100,000; however, the true incidence may be much higher [4]. Although NCSE presents with various symptoms, it is much more difficult to distinguish NCSE onset compared to convulsive epilepsy.

In our patient, we observed transient thyroid-stimulating hormone (TSH) elevation following NCSE that was misdiagnosed as subclinical hypothyroidism. After administration of levothyroxine, our patient experienced levothyroxine-induced NCSE. In the present report, we consider the possibility of TSH elevation following NCSE, and the possibility of levothyroxine superinducing NCSE.

## Case presentation

Our patient was a 68-year-old Japanese woman who had no history of major illness. Nine years before her admission, she had been taking daily triazolam prescribed by a local doctor for insomnia, and four years before admission, she attempted to sleep without taking this medication but was unable to. The next day, her conversation was unfocused and she was unable to relax. She was examined at our hospital's neurology department on the same day. Our patient seemed normal in appearance and showed no signs of automatism or convulsions. However, she exhibited marked disorientation and a euphoric mood. Her free tri-iodothyronine and free thyroxine (fT3 and fT4, respectively) levels were normal but her TSH levels were high (10.88 μIU/mL), and she was accordingly admitted to the neurology department with suspected Hashimoto's encephalopathy. However, our patient tested negative for anti-thyroid antibodies (A-TPO: Anti-Thyroid peroxidase, A-TG: Anti-Thyroglobulin, and A-MC: Anti-Microsome), her cerebrospinal fluid appeared normal, and there were no abnormalities on a thyroid ultrasound scan; therefore, her results did not support the diagnosis of Hashimoto's encephalopathy. Her EEG results showed continuous, irregular, generalized 3 Hz spike-and-wave complexes, and our patient was accordingly diagnosed as having NCSE (absence status epilepticus). After administration of 5 mg of diazepam intravenously, the 3 Hz spike-and-wave complexes disappeared. The next day, her orientation improved. She was discharged after 11 days and began taking zonisamide (200 mg/day).

Three years before admission, she experienced depression, which resolved with flunitrazepam alone. One year before admission, because her speech was slightly confused, she was investigated at an internal medicine clinic and was found to have a TSH level of 5.7 μIU/mL and an fT4 level of 1.0 ng/dL. At this time, our patient was diagnosed as having subclinical hypothyroidism and given 25 μg/day of levothyroxine. However, she did not adhere to this medication until 12 days before this admission.

**Figure 1:**
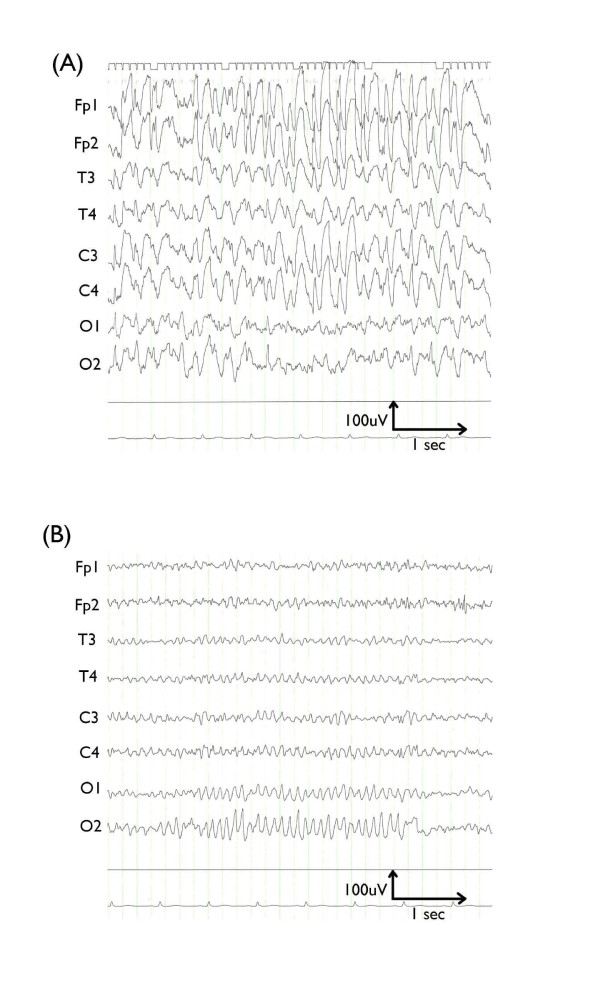
**Electroencephalograms taken on the first (A) and second day (B) of hospitalization**. On the first day of hospitalization, a general absence of normal brain waves with continuous irregular 3 Hz to 5 Hz spike-and-wave complexes is apparent. On the second day of hospitalization, a large number of alpha waves with a basic frequency of 10 Hz were seen, predominantly in the occipital area. There was no evidence of paroxysms.

Our patient's husband became concerned because our patient, who was always methodical in her behavior, was forgetting to close gas valves and switch off the lights. Our patient was examined at our facility and reported having circumstantial thinking, but no other neurological symptoms. However, she was unable to remember her behavior from that morning. We suspected that our patient was suffering from a mild deterioration of consciousness and performed an EEG. This showed continuous 3 to 5 Hz polyspike-and-wave complexes (Figure 1A), and our patient was hospitalized with NCSE.

On admission, a physical examination revealed no non-pitting edema, or any irregularities in the central nervous system, motor system, sense of balance, reflexes, sensory systems, or in the head, neck, or spine. Her biochemical and hematological findings were unremarkable. Endocrine analysis revealed the following: TSH 11.19 μIU/mL, fT4 1.04 ng/dL, fT3 2.61 pg/mL and prolactin (PRL) 30.74 ng/mL (her TSH and PRL levels were hence considerably elevated). A cranial MRI scan showed no gross lesions, and a pituitary MRI scan revealed no tumors. After admission, we supplemented her medication with a 10 mg/day diazepam suppository and 0.5 mg/day of oral clonazepam. Our patient's level of consciousness had greatly improved by the next day after this admission, and all evidence of paroxysmal discharge had disappeared (Figure 1B). Four days after this admission, further endocrine testing results showed normal levels of TSH (3.575 μIU/mL) and her PRL level had fallen (16.18 ng/mL). To determine the cause of the high TSH levels detected on admission, thorough endocrine examinations including thyroid ultrasound and anti-thyroid antibody tests were performed at our Division of Endocrinology, but there was no evidence of thyroid disorder. We therefore decided that in the absence of thyroid disorder, and because of the potential for further NCSE episodes, our patient did not require levothyroxine. Our patient was discharged from our department 12 days after this admission. There have been no further signs of NCSE recurrence to date.

## Discussion

**Figure 2:**
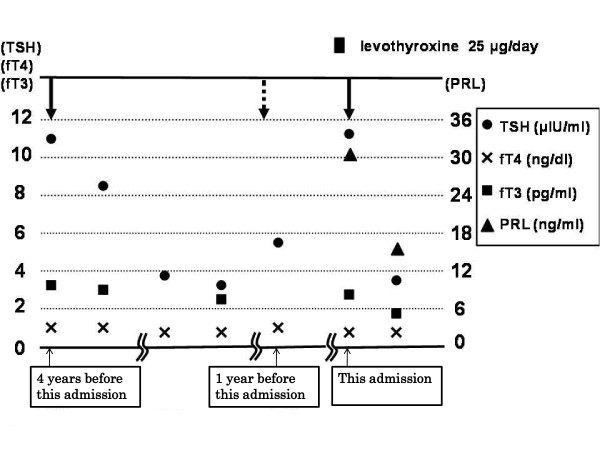
**Thyroid hormone and prolactin levels over the course of the illness and follow-up**. Arrows indicate the dates on which non-convulsive status epilepticus (NCSE) was confirmed by electroencephalography. Broken arrows indicate dates of possible NCSE identified by clinical factors but not confirmed through electroencephalography. The black square at the top of the figure indicates the period in which levothyroxine was taken.

In our patient, endocrine testing revealed elevated TSH levels associated with NCSE while fT3 and fT4 levels remained normal. However, thyroid function tests performed after both episodes (unquestionably NCSE) showed no evidence of a thyroid disorder. Our patient was monitored without further treatment and her TSH levels normalized (Figure 2). The details of pathogenesis are unknown; we believe that the etiology of the first episode of NCSE might be the intermittent use of triazolam. During the course of her illness, our patient showed confusion and was unfortunately misdiagnosed as having subclinical hypothyroidism based on findings of endocrine testing at another clinic and was administered levothyroxine. The episode of NCSE a year before this admission was induced without levothyroxine. Non-compliance with zonisamide medication, inadequate therapeutic levels of the zonisamide medication, and the intermittent use of benzodiazepines may have exacerbated this NCSE recurrence. However, the etiology of the NCSE recurrence on the day our patient was hospitalized in our department was also uncertain, and may have occurred after prescribing levothyroxine. We hypothesize that the high TSH levels were caused by NCSE, and that levothyroxine induced NCSE recurrence.

Several cases have been reported where epileptic discharges induced endocrine abnormalities. Prolactin has been reported to increase following epileptic seizures [5], as have TSH levels. Apria *et al*. [6] reported that the pituitary gland can suddenly release TSH following electroconvulsive therapy. In addition, it is reported that ictal epileptic activity in the mesial temporal structures may propagate to the hypothalamus [7]. Our patient had diffuse electrical discharges in the brain including the bilateral temporal lobe (Figure 1A), therefore NCSE might feasibly have induced elevation of TSH and PRL levels.

However, it should be verified whether physicians with little experience of NCSE are likely to start levothyroxine when they encounter patients whose laboratory data are similar to the findings from our patient. A review of studies on latent thyroid disorder by Surks *et al*. recommended treatment with levothyroxine for women aged 60 and older with TSH levels in the 4.5 to 10 μIU/mL range [8]. This recommendation strongly supports the decision to use levothyroxine in our patient's case. However, though NCSE is rare, if it can induce abnormal levels of TSH, it is important to evaluate cognitive symptoms in detail.

In addition, there are several reports proposing a negative correlation between thyroid function and epileptic threshold. Jabbari and Huott reported a case of concurrent hyperthyroidism and epileptic seizure; as thyroid function improved, so did epileptic seizures and abnormal brain wave patterns [9]. Maeda and Izumi reported on a patient who developed generalized convulsions emerging concurrently with Graves' disease [10]. In the case of our patient, therefore, it seems that levothyroxine likely lowered the epileptic threshold, which provoked the NCSE before our patient was admitted to our department.

NCSE-induced hormone abnormality resulting in TSH elevation with normal fT3 and fT4 levels may be misdiagnosed as subclinical hypothyroidism. Particular attention is required because treatment of subclinical hypothyroidism with levothyroxine may result in a lowered epileptic threshold and consequent NCSE recurrence. Because some cases presenting with a high TSH with normal fT3 and fT4 levels may have NCSE as an underlying cause, particular caution is necessary when interpreting these particular endocrine abnormalities.

## Conclusions

We present a case of TSH elevation misdiagnosed as subclinical hypothyroidism following non-convulsive status epilepticus. In this case report, we suggested the possible mechanisms of latent hypothyroid-like hormone abnormality following epileptic seizures and the possibility of provoking epileptic seizures by administering levothyroxine for misdiagnosed subclinical hypothyroidism.

## Consent

Written informed consent was obtained from the patient for publication of this case report and any accompanying images. A copy of the written consent is available for review by the Editor-in-Chief of this journal.

## Competing interests

The authors declare that they have no competing interests.

## Authors' contributions

AW wrote the first draft of the manuscript. YS, ST, YK, HY, and SN provided helpful comments on the draft of this paper from a psychiatrist's viewpoint. SM provided helpful comments on the draft of this paper from an endocrinologist's viewpoint. All authors read and approved the final manuscript.
